# Beneficial laggards: multilevel selection, cooperative polymorphism and division of labour in threshold public good games

**DOI:** 10.1186/1471-2148-10-336

**Published:** 2010-11-02

**Authors:** Gergely Boza, Szabolcs Számadó

**Affiliations:** 1 Department of Plant Taxonomy and Ecology, Institute of Biology, Eötvös University, Pázmány Péter s. 1/c, H-1117 Budapest, Hungary; 2 HAS-ELTE Research Group for Theoretical Biology and Ecology, Institute of Biology, Eötvös University and the Hungarian Academy of Sciences, Pázmány Péter s. 1/c, H-1117 Budapest, Hungary; 3 Collegium Budapest, Institute for Advanced Study, Szentháromság u. 2, H-1014 Budapest, Hungary

## Abstract

**Background:**

The origin and stability of cooperation is a hot topic in social and behavioural sciences. A complicated conundrum exists as defectors have an advantage over cooperators, whenever cooperation is costly so consequently, not cooperating pays off. In addition, the discovery that humans and some animal populations, such as lions, are polymorphic, where cooperators and defectors stably live together -- while defectors are not being punished--, is even more puzzling. Here we offer a novel explanation based on a Threshold Public Good Game (PGG) that includes the interaction of individual and group level selection, where individuals can contribute to multiple collective actions, in our model group hunting and group defense.

**Results:**

Our results show that there are polymorphic equilibria in Threshold PGGs; that multi-level selection does not select for the most cooperators per group but selects those close to the optimum number of cooperators (in terms of the Threshold PGG). In particular for medium cost values division of labour evolves within the group with regard to the two types of cooperative actions (hunting vs. defense). Moreover we show evidence that spatial population structure promotes cooperation in multiple PGGs. We also demonstrate that these results apply for a wide range of non-linear benefit function types.

**Conclusions:**

We demonstrate that cooperation can be stable in Threshold PGG, even when the proportion of so called free riders is high in the population. A fundamentally new mechanism is proposed how laggards, individuals that have a high tendency to defect during one specific group action can actually contribute to the fitness of the group, by playing part in an optimal resource allocation in Threshold Public Good Games. In general, our results show that acknowledging a multilevel selection process will open up novel explanations for collective actions.

## Background

The intriguing phenomenon of cooperation has fascinated experimental and theoretical researchers for decades, as it is essential for understanding the complexity of life, and life itself [1-5]. A dilemma derives from the fact that selfish individuals have an advantage over those who act cooperatively, by which we mean a costly act that can benefit others [1,6,7]. Although selfish 'defectors' interacting with their own kind have a lower 'payoff' than cooperators, defectors would eventually totally replace cooperators in the population [8]. However a wide variety cooperative behaviour can be observed in nature [7,9]. Moreover, it is a common observation that humans [10-12] and some animal populations such as lions (*Panthera leo*) [13,14] are polymorphic. Individuals that have a high tendency to cooperate live together with those that have a high tendency to defect. The proportion of these 'laggards' can be high in certain societies and they are not being punished, as in the case of lions [14] or in a number of human hunter-gatherer societies [11,15]. Observations suggest that the average tendency for cooperation in these populations appears to be stable in the long term. This is a perplexing set of observations, not yet fully understood. Existing explanations rely either on the snowdrift game [16,17] with homogeneous population structure, punishment [18], or spatially explicit population structure [19]. None of these explanations can be applied perfectly here as for example lion and human populations are neither completely well-mixed, nor spatially explicit in a sense as sedentary organisms, and punishment is also not common in these instances.

Majority of the current evolutionary game theoretical studies that shed light on the mechanism behind many cooperative phenomena in biological systems concentrated on pair-wise interactions between individuals [3,6-8,10,16,20][but see for e.g. [21]]. Arguably numerous examples of group interactions found in nature however can be described as *n*-person games [22-25,21], in which more than two 'players' interact with each other at the same time in the form of group actions [26-30]. During group interactions individuals typically invest into common goods or common goals [31]. In most of the cases this is available for everyone, that is the common good is non-monopolizable (or non-excludable, non-exclusive) and non-rival (synonym of joint in supply, non-diminishable) [32-35]. This raises a collective action problem, where non cooperators, often termed as free riders, can reap the benefit of the common good without investing into it [24,34,35].

**Figure 1:**
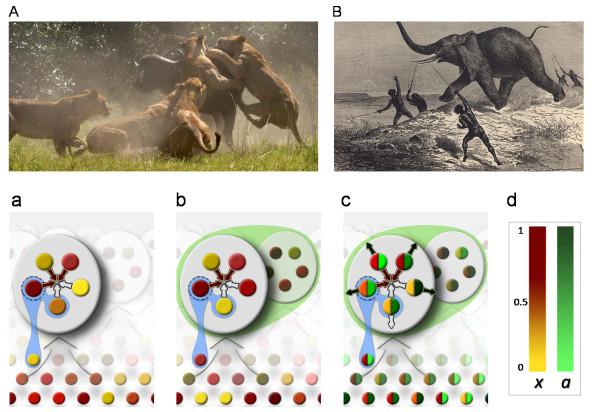
**The schematic representation of the relationships between the different units in the different model scenarios**. Lion hunting groups (**A**) and human hunters (**B**) successfully trap large preys by encircling it, that solitary or few individuals would be unable to do so. The presented model based on these remarks, where individuals are represented by the coloured circles, which indicate their trait composition (**a-c**). The individuals' location in the population can be fixed in the spatially explicit model, or individuals can disperse randomly in the well-mixed model. The group (big grey circle) is composed of individuals existing at a location of certain size at that moment (grey arrow). Individuals make their contributions to the group hunting effort (small green arrows), and receive the payoff according to the rules of the game. (**a**) Individual competition only. A randomly chosen focal individual (dashed circle) competes either with one of its group mates in the spatially explicit model, or with a randomly picked individual from the population in the well-mixed model (blue shadings). (**b**) Individual and group competition. Individual competition as before, and after the focal group competes with either a neighbouring group or a random group from the population (green shading). (**c**) Individual and group competition with voluntary participation in the both of the group actions, such as the cooperative hunting and the competition between groups for territories. (**d**) In the last case individuals have two continuous traits which determine their propensity to participate in the group actions, *x *for the Public Good Game, and *a *for the group competition game, both between 0 and 1. (pictures from: classicafrica.com; gtemporium.wordpress.com).

In many cases of a collective action the achieving the group goal, for example capturing prey depends on the number of encircling hunters [36,37], and not necessarily on their individual efforts (Figure 1). During numerous instances of cooperative hunting several individuals simply fill the position of blocking, diverting, flushing the prey. This threshold effect is documented for cooperative hunting situations in various social carnivores including lions [36,37], African wild dogs (*Lycaon pictus*) [38-40], chimpanzees (*Pan troglodytes*) [41], Harris' Hawks (*Parabuteo inicintus*) [42] and humans [43,44]. For example when lions are hunting small prey, each lion pursues its own animal. However with larger, faster or more difficult prey [45] the cooperation of a group of hunters is needed to encircle, split the herd, to spot, surround and kill the animal [36,37,46]. In order to make the hunt successful several lions have to work together (about 4-7) [36,37,47,48], get close to the prey before starting the attack (30-50 m) [36,37,46,49] and cut off its escape routes by encircling the prey from different points and filling the roles of "centers" and "wings" [36,37]. Below a threshold group size it is not only hard to capture the prey but to defend it against hyenas [50]. Chimpanzees [41,51] or wild dogs [39] sometimes use similar encircling tactics. Harris' Hawks either perform surprise attacks on lagomorphs from different directions, or use flush-and-ambush tactics to capture hidden prey [42]. Some other bird species also hunt cooperatively larger prey, such as Brown-necked Raven (*Corvus ruficollis*) on Egyptian Mastigure (*Uromastyx aegyptius*), in which case two birds fill the tasks of blocking the escape routes of the prey while several others attack it from several directions, always performing the direct attack on the most exposured part of the lizard [52]. Many sea mammal species, such as killer whales (*Orcinus orca*), humpback whales (*Megaptera novaengliae*), bottlenose dolphins (*Tursiops truncatus*) and spotted dolphins (*Stenella frontalis*) chase school of fish into a tight ball, sometimes driving it into a rock or to the water surface with coordinated group action, some performing tail slaps, creating a curtain of air around the fish ball or performing feeding bouts [27,53-56]. Moreover some whales may use their acoustic "trumpets" creating a "wall sound" combined with the bubble net, as recently suggested by Leighton and colleagues, to trap the fish inside the curtain [57]. Cooperative hunting in human hunter-gatherers societies has been common, as coordinated action of several hunters was necessary to chase larger preys into a natural trap and to hunt them down (consider for example the buffalo jump sites) [58,59]. There are numerous examples of threshold effects in the operation of modern human societies and in economics [60]. Take as an example the requirement of a minimum number of hunters on the boat during traditional whale hunting [44] or the necessity of collective mass for successful employee strikes [61,62]. Finally, threshold effects in group cooperation can appear not only in human and animal hunting societies. There are well documented examples of a lower group size threshold in some cooperatively breeding species [40,63,64]. Evidence from microbial cooperation suggests, that in some cases a threshold number of cooperators, that is bacteria which produce extracellular chemical components, is crucial in producing a public good [65,66]. Also, at the dawn of life the first protocells were most probably composed of cooperative elements, RNA molecules, which could have controlled the metabolism of the protocell acting like enzymes, and the complete metabolism most probably required the cooperation of sufficient number of elements, or at least the elements of an autocatalytic core [5,67].

Models of Public Good Game nicely capture the main features all of the above described cooperative phenomena. But the traditionally used linear benefit return function is insufficient in capturing the threshold effect for the optimal number of individuals that is necessary to perform the given group action, as close to the threshold joining one or few more cooperators disproportionally increases the success of the group action [43,68,69]. So instead a nonlinear return paradigm is more appropriate [68,70-72], such as in the *n*-player Threshold Public Good Game [71]. In this version of the Public Good Game (PGG) a successful cooperative effort is achieved only if the number of cooperating individuals reaches a given threshold, just like in the above described situations. Different levels of cooperation can be evolutionary stable outcome in such games, depending on the cost of cooperation, and the proportion of initial cooperative decisions in the population [71].

While these situations assume that groups of individuals engage in an interaction which may or may not end in successful cooperation these groups themselves often compete with each other [35]. For example, in one of the most studied group behavioural biological systems lioness form a pack to hunt together yet they are in direct competition with other lion packs with which they share common borders [13,14,47,73,74]. The same holds for all group hunting territorial species from hyenas through whales, African wild dogs to humans [75-78].

Here we study the interaction of selection acting on the level of individuals engaged in a threshold PGG and selection acting on these groups while competing for territories. Thus, our model differs from previous models of TPGGs [71,72] and that of multi-level selection [77] by explicitly integrating these two components. We do so first by giving analytical solutions for the evolutionarily stable level of cooperation for various group sizes and threshold levels at first assuming only individual selection; then by studying the interaction of individual and group level selection with a series of computer simulations validated by the numeric results. We study 3 basic setups in the computer simulations: (i) individual selection only (Figure 1.a), (ii) group selection only, (for comparison), and (iii) the combination of both, in which case first we assume that (iii/a) all individuals are obliged to participate in group defense (Figure 1.b), then we relax this assumption by allowing (iii/b) voluntary participation (Figure 1.c). Individual selection in our model allows individuals to compare their success with other individuals, or compete for resources, while group selection based on the idea that stronger groups may outcompete weaker ones by overtaking their territories. The structure of the game is described in the Methods section.

## Results

### Effect of different threshold functions

**Figure 2:**
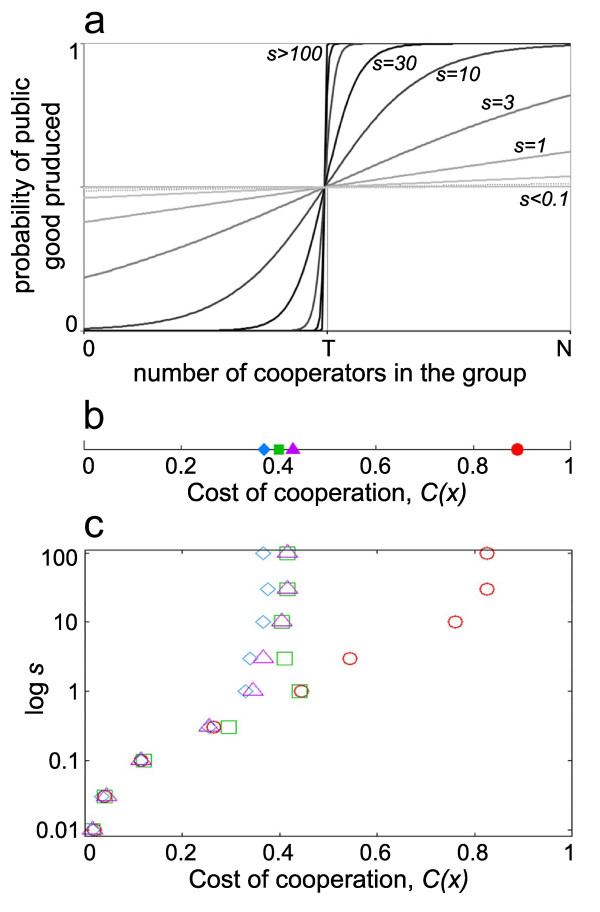
**The effect of *s *parameter on the level of cooperativeness in the population**. (**a**) The *s *parameter determines the steepness, hence the shape of the sigmoid function. When *s *approaches ∞, the probabilistic benefit function approaches to strict deterministic step-wise function, meaning that above the threshold (*T*) the public good is always achieved, while below never. With decreasing *s *the probability of public good achievement changes from strict all or nothing to a smoother function. In the later case there is a non-zero probability of achieving the common goal even below the threshold, and also above the threshold the group can fail. (**b**) The position of the hysteresis point with the use of strict deterministic step-wise benefit function. (**c**) The effect of *s *(steepness of the sigmoid function) parameter on the location of the hysteresis point. The hysteresis point indicates the highest cost value for which cooperation is still a stable outcome of the game, and even a small increase in the cost would cause the collapse of this polymorphic equilibrium to defection. Below this cost value we always find cooperative equilibria. (●,○: T = 5; ■,□: T = 4; ♦,◊: T = 3; ▲, Δ: T = 2).

First we study the effect of the benefit function shape on the level of cooperation in the population (Figure 2) by means of IBM simulations (see Methods). For this we substitute the threshold benefit function with a sigmoid function (s-shape function), which gives the probability of achieving the group goal, that is receiving the benefit for different levels of cooperative effort. A good indicator of the cooperativeness in the system is the position of the hysteresis point [71], which above the proportion of cooperators drops to zero in all cases. Below the hysteresis point, we always find a stable level of cooperation, while if the cost of cooperation moves above the hysteresis point the population rapidly evolves to zero cooperativeness. The positions of the hysteresis points for different threshold values assuming sigmoid functions (Figure 2.a) don't change significantly for a wide range of *s *parameter (steepness of the benefit function). For high values of *s *(1 <*s *< 100), the hysteresis point appears at the same cost values (*C(x)*) for cases T≠N (Figure 2.c), as with the strict deterministic step-wise function (Figure 2.b). For T = n, this parameter range is narrow (10 <*s*), and a slight change in the steepness of the benefit function results in major changes in the cooperative equilibrium. Our results indicate that the dynamics of the system remains the same for relatively steep sigmoid functions than for a step-wise function. In the following we will use the later one in our simulations.

### Results for individual level selection

**Figure 3:**
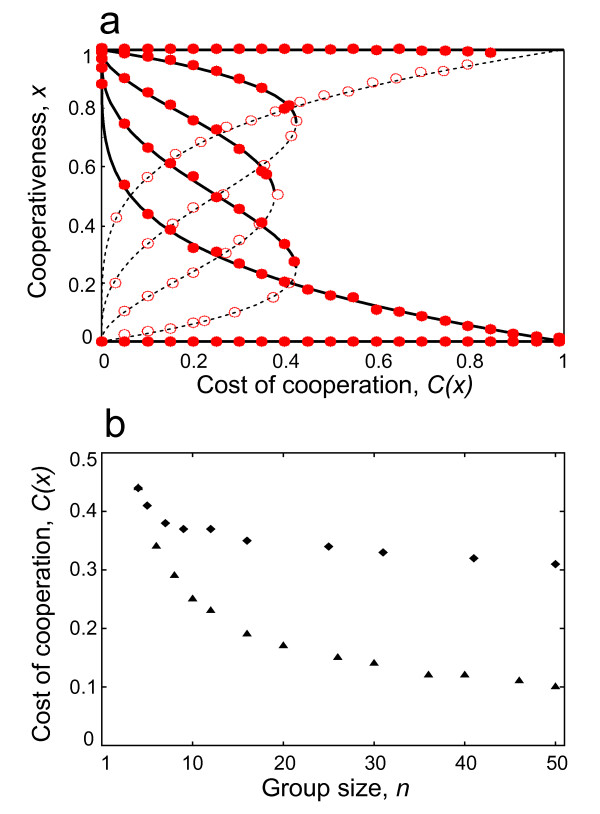
**Stable and instable fix points of the model and the position of the hysteresis point for different group sizes (*n*)**. Results from numerical and individual-based (IBM) simulations show the same results. Instable fix points (dashed line for numerical simulations, open red circles for IBM simulations) separate the interior stable fix points (thick lines for numerical and filled red circles for IBM simulations) and 0 cooperativeness in the system. **a**, Group size (*n*) is 5, the threshold values are (*T*) 5, 4, 3, 2, 1. **b**, the locations of the hysteresis points (i.e. the maximal cost where cooperation still can be a stable), with different group sizes (*n*). (♦: *T *= *n*-1; ▲: *T *= *n*/2).

Next we employ the method described in Bach *et al*. 2006 [71] to find the stable and instable equilibria of the model for individual selection only (see Figure 1.a; further details in the Methods). We also run a series of simulations in our individual based model (IBM) for comparison with the analytical model (i.e. to "calibrate" the IBM simulations). The equilibrium level of cooperation depends both on the size of the group (*n*) and on the threshold level (T) (Figure 3). Figure 3.a depicts the resulting fixed points of the system for given group size and for increasing *T*, from the analytical model (solid curves for stable and dashed curves for instable fix points), and from individual based simulations (dots), which show a close fit. The higher the threshold value the higher is the ratio of cooperators at a given group size (Figure 3.a). Not surprisingly the highest level of cooperation can be achieved when the threshold value equals the size of the group, and for small group sizes, the average level of cooperation is higher (Figure 3.b). The unstable fix points separate the attractors of the interior stable fixed points of cooperation from the attractors of zero cooperativeness (Figure 3.a). Above the separatrix, cooperation prevails, below this boundary, cooperation diminishes. All results from IBM model show a perfect fit for the predictions of the analytical model.

### Introducing multilevel selection

**Figure 4:**
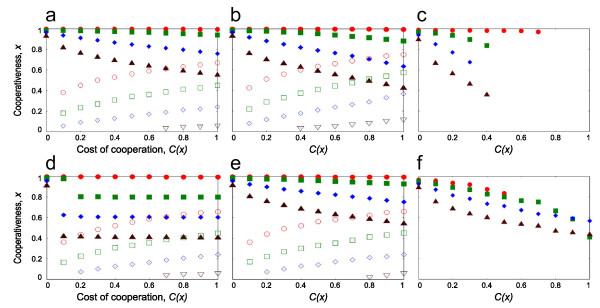
**Equilibrium frequency of cooperators as a function of the cost of the Public Good Game (*C(x)*)**. The panels depict the results of the individual-based simulations for non-zero stable fix points (filled marks) and instable fix points (open marks). The groups are composed of 5 individuals either picked randomly from the population (**a**, **b**, **c**), or from the same site in the model with spatial population structure (**d**, **e**, **f**). In cases **a**, **d **competition occurs only between groups, in the simulations of **b **and **e**, both individuals and groups compete with each other with compulsory participation. Finally in the cases of **c**, **f **both individual and group level selection are present and participation in the group stage is voluntary. The different marks are depicted to different threshold values (●,○: *T *= 5; ■,□: *T *= 4; ♦,◊: *T *= 3; ▲, Δ: *T *= 2).

In the following we explore the individual based model with well-mixed and spatial population structure. In the first step, we introduce group level selection to the individual based model (see Figure 1.b) keeping the participation in the group defense compulsory. Figure 4 depicts the cases of only group selection (a,d), and the combination of group and individual selection when group defense is compulsory (b,e). The most striking effect of introducing group level selection is that the hysteresis effect disappears in all cases. In the well-mixed case (Figure 4.a) the level of cooperation is higher than without group selection, however, aside from the case *T *= *n*, the population is still polymorphic. It means that groups with higher number of cooperators do not necessarily enjoy a competitive advantage. Groups in general respond in proportional manner to the threshold value, i.e. they optimize resource allocation. This can be best seen from the spatially explicit simulation with only group selection (Figure 4.d) as in this case the average equilibrium level of cooperation is exactly the threshold level. This implies that any deviation from the optimal group composition results in a disadvantage for the group, not only if the number of defectors is higher, but also if the ratio of cooperators and defectors differs from the optimal in any ways (i.e. groups that optimize their allocation this way have higher fitness than groups that do not, see for further details in Additional file 1 figures from S.3 to S.4). Introducing individual level selection decreases the level of cooperation in the well-mixed case (Figure 4.b vs. 4.a), however interestingly and somewhat counter intuitively it increases the level of cooperation in the spatially explicit case (Figure 4.e vs. 4.d). Equilibrium populations are still polymorphic aside the case *T *= *n*. Figure 4.c and 4.f depicts the case when participation at the group competition stage is voluntary. When group defense is cost-free (*C*(*a*) = 0) the level of cooperation in the Public Goods Game is the same as in the previous cases (result not shown). When group defense is costly (*C*(*a*) = 1) then the level of cooperation in the PGG is lower (Figure 4.c and 4.f) and the hysteresis effect reappears in the well-mixed case (Figure 4.c), but not in the spatially explicit model (Figure 4.f). Note, however, that when cooperation is present the polymorphic nature of the equilibria is preserved (aside *T *= *n *in the wellmixed model), that is, resource allocation is still optimized, groups with higher number of cooperators do not win out by default.

**Figure 5:**
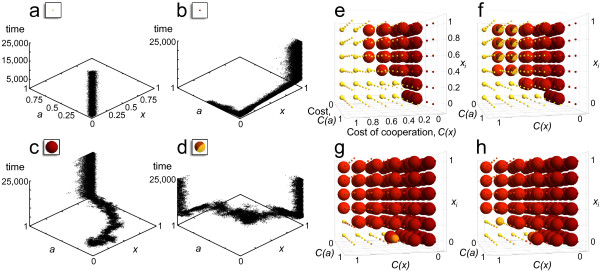
**Simulation results for the multilevel selection model with voluntary participation**. The average propensity for cooperation concerning the two kinds of group actions, the public goods game and the voluntary group competition action as a function of the two costs of cooperation (*C*(*x*) and *C*(*a*)) and the initial ratio of cooperators (*x_i_*) in the Public Goods Game. (**a**) For high cost values the tendency of cooperating in both of group actions is low, (**b**) or there is a full cooperation in the Public Goods Game, but full defection in the group defense action. (**c**) If the costs of cooperation are not high, every individual cooperates in both of the group actions (*x *= ~1, *a *= ~1). (**d**) At the boundaries of these regions, for intermediate cost values division of labour evolves in the population (**h**). On graphs **e**.-**h. **each bubble illustrates the results of an individual based simulation, the size representing the average *a *in the population (large bubbles represent *a *= 1 and vice versa), and the colouring depicting the average value of *x *(red bubbles denote *x *= 1, while yellow ones denote *x *= 0). For the simulations we either used no initial incentive in the populations for participating in the group competition (*a_i _*= 0) (**e**, **g**), or the simulation was started with full participation (*a_i _*= 1) (**f**, **h**). The 5 group members were either picked randomly from the population in the well-mixed model (**e**, **f**), or from the same site in the spatially explicit model (**g**, **h**). ((**a**) *C*(*x*) = 0.5, *C*(*a*) = 0.5, *x_i _*= 0, *a_i _*= 0; (**b**) *C*(*x*) = 0, *C*(*a*) = 0.6, *x_i _*= 0, *a_i _*= 0.5; (**c**) *C*(*x*) = 0.2, *C*(*a*) = 0.05, *x_i _*= 0.05, *a_i _*= 0.2; (**d**) *C*(*x*) = 0.6, *C*(*a*) = 0.2, *x_i _*= 0.6, *a_i _*= 0.5).

Finally Figure 5 depicts the results as a function of the two kinds of costs and the initial number of the cooperators in the PGG when both the participation in the hunt and in the group defense is voluntary (see Figure 1.c). We can observe four types of dynamics in our simulations (Figure 5.a-d). If the costs of cooperation (*C(x)*, *C(a)*) are high, individual willingness to cooperate evolves to zero (Figure 5.a). Individuals willingly cooperate in group defense (i.e. large bubbles, that is high values of *a*, close to 1) only if there are high levels of cooperation with regard of group hunting (i.e. the colours of the large bubbles ranges from orange to red (~0.7-1) on Figure 5.e-h, Figure 5.c). However, cooperation in the Public Goods Game can stay at high levels even if the propensity to participate in group defense is low (i.e. there are small red bubbles when the cost of group defense is high) (Figure 5.b). Polymorphism of different cooperative efforts in the Public Goods Game is still present at many of the equilibria (i.e. lighter shade of red), and interestingly division of labour evolves at medium values of costs in the well-mixed case (Figure 5.f red/yellow bubbles). In these cases, polymorphism occurs on the population level, where individuals cooperate strictly in one of the collective actions, but never both (Figure 5.d).

Our results also indicate, in line with the general conclusion of previous models that spatial structure favours cooperation [6,8,19,79], as high values of cooperation evolve for both public actions (hunting and group defense, see large red bubbles) even at high cost values (Figure 5.g and 5h). We observe division of labour in the spatially explicit model only under given circumstances (see Additional file 1 for further details), which suggests that this outcome is unstable in these cases with small group size. Note that when *C(x) *> 0 cooperation evolves only if the initial proportion of cooperation is not zero in the population with well-mixed structure, which indicates a separatrix in the system. However, spatial population structure promotes cooperation and it allows cooperators to invade at higher cost values with regard of both types of costs (i.e. compare 5.e with 5.g and 5.f with 5.h).

## Discussion

Here we show that multilevel selection in threshold PGG can maintain stable levels of polymorphism (i.e. a stable mixture of cooperators and defectors) without the need of punishment or spatially explicit population structure. We give a primary demonstration that the described dynamics holds not only for step-wise benefit function (strict threshold function), but for a wide range of sigmoid curves between the step-wise and the linear benefit functions (s-shape function). Our results further indicate that polymorphism in group hunting and defense can be adaptive in case of multilevel selection, and to our knowledge, we provide a pioneer report on the division of labour in multiple Public Good Games. We conclude that what was regarded as cheating at the individual level is in fact can play a significant part in the optimization at the group level, which optimization is provided by the described mechanisms working on behavioural polymorphism.

Many of the collective actions produce goods that are non monopolizable, meaning that no one can be excluded from benefiting [33-35], thus free riders can exploit this collective good without paying the cost of production [28]. Threshold effects in Public Good Games can provide an often neglected, yet powerful explanation for the observed polymorphism in the population, as an alternative to punishment [18], spatial structure [19] or various payoff functions [16,17].

Population level polymorphism is a stable outcome in Threshold Public Good Games, where cooperators and free riders stably live together in the population [71,72], as long as the cost of cooperation doesn't exceed a limit cost value, the hysteresis point. Our model predicts that for an increased group size this hysteresis, that is a sudden drop from cooperation to defection, appears at lower cost values, compared to smaller groups (see Figure 3.b). To put it in a different way, with large groups cooperation can be maintained only if the cost of cooperation is relatively low. Also for higher cost values the instable fix points move closer to the stable fix points, which means that the invasion of cooperators in a population of defectors becomes harder, while the invasion of defectors into a cooperative population becomes more likely.

Introducing multilevel selection into the model is a logical step towards reality, as in many biological and human examples where collective hunting occurs intergroup conflicts can be also observed [35,74-77]. When group level competition introduced explicitly into the model population level polymorphism is stabilized both in well-mixed and spatially explicit populations. Thus, multilevel selection need not select for the groups with the highest number of cooperators as it is often assumed. Groups that optimize the number of hunters enjoy an advantage over those groups that hunt (cooperate) on a higher, unnecessary level. Thus, defection of a given proportion of individuals during one specific cooperative group action can be an adaptive strategy for the group depending on the cost values. Because group level performance (i.e. success in the PGG) determines both individual and group level success any deviation from the optimal group composition would cause a disadvantage in some way or other. If the number of cooperators is lower than the threshold value then the collective action (i.e. hunting) is unsuccessful, thus neither cooperators nor defectors gain anything, and these groups are being replaced by successful groups. On the other hand, if the number of cooperators is higher than what is required for providing benefit for the whole group (in our example capturing the prey), the energy loss of unnecessary effort would cause a disadvantage when the group faces a conflict (fight for territory). Hence our novel result is that laggards, who were previously seen as exploiters of the common goods provided by cooperators [13,14], do actually contribute to the fitness of the group by keeping the group level allocation at the optimum level.

Our results also verify that if participation in two distinct collective actions which produce shared benefits is costly, such as hunting and territory defense, then selection pressure on cooperators can result division of labour to evolve, predicting that it will evolve only for a narrow range of cost values. Interestingly, behavioural polymorphism first appears at the individual level, that is all of the individuals participate in both collective actions with intermediate probabilities. However, this state is not stable and evolution drives the system towards division of labour at the population level, where individuals mostly participate only in one of the collective actions (i.e. polymorphism appears at the group level). This result is robust in our simulations at medium cost values, and turns out to be stable on the long term. Experimental support for this context dependent role specialization is poor yet [but see[56,80]], however the idea that cooperators and 'free riders' switch roles in different contexts is not obscure [28,35,56,81,80], as long as there is a trade off situation between two energy consuming group actions [81].

Our model has a simplistic assumption, that individuals have only two heritable traits that describe their behavioural decisions, this two are sufficient for maintaining polymorphism in the population. Obviously regularly many genes effect behaviour, which explains higher polymorphism, still there is evidence suggesting that some may play disproportionally important role in behavioural switches [82]. Accordingly, our model can provide a potential explanation for the observed polymorphism in lions in threshold game like situations [36,37,46] and the presence of laggards in the population [14,83].

The importance of intra group conflict in human evolution seems to gain a new currency [77,78], however since the technology of hunting humans changed a lot in the last 250,000 years (i.e. evolution of spear points, bows, arrows, etc. [84,85]), it is more difficult to evaluate whether a threshold effect existed in hominid plio-pleistocene group hunting. The conception of hunting large preys would inevitably suggest so and the fact that hunting of medium-sized and large ungulates started long before stone-tipped and bone-tipped weapons were widely used strongly suggests that cooperation amongst hunters was essential for the capture of large games [85]. If it did so, then our model applies and has the potential to explain the observed polymorphism in humans as well. Interesting implication of our results is that once human societies become larger and more fluid in composition this polymorphism was no longer necessarily adaptive and definitely was not looked upon as desirable. This, in turn could have triggered the evolution of cultural norms and institutions that attempts to obtain an universally high level of cooperation from all the members of the society regardless of their predispositions.

## Conclusions

Here we present a multilevel selection model of interdependence in group living species, where cooperation is modelled as an *n*-player Threshold Public Good Game and where selection acts both at the individual and at the group level. We have found that the population can evolve into stable levels of cooperative polymorphism, where cooperators and free riders -laggards- can stably live together. Our results indicate: (i) that the described dynamics holds for a wide range of probabilistic sigmoid benefit functions, not only for strict deterministic step-wise function; (ii) that multilevel selection need not select for the highest number of cooperators within groups but instead it may selects for polymorphism depending on the details of the TPGG; (iii) that defectors contribute to the group fitness as much as they help the group to achieve the optimal amount of investment at the group level; (iv) that division of labour might evolve with regard of the participation in the two collective actions for medium cost values of cooperation; (v) and that spatial population structure promotes cooperation in TPGGs.

## Methods

### The game

The structure of the game is as follows: individuals of a group can engage in a cooperative activity (i.e. hunting, resource purchase) where every individual can play two strategies, either to cooperate with probability *x *or to defect with probability 1-*x*. The cooperative players join in the group effort (group size is *n*), and thus pay a fix cost of cooperation (*c*). In contrast, defectors do not pay the cost of the game. If the number of cooperators is equal or above a given threshold value (*T*), then all of the individuals within the group can acquire the benefit (*b*) of cooperation regardless whether they cooperated or not. This derived from the non-rival, non-excludable features of the game [33-35], that is, no individual can be excluded from the acquired benefit of the hunt, and each consumer gains the same proportion without depleting it. However if the number of cooperators does not reach the necessary number which is required for the successful achievement of the group action then no one gets the benefit, but cooperators still pay the cost. The fitness (*W*) is calculated as the benefit minus the cost of the game.

### Analytical solutions

We use the method described in Bach *et al*. 2006 [71] to find the stable and instable equilibria of the model. According to the Bishop-Cannings theorem [86] strategies supporting a mixed equilibrium need to have the same fitness. Thus, at a mixed equilibrium supported by cooperators and defectors the fitness' of both strategies should be the same. Let us denote the level of cooperativeness in the population as *x*, then the fitness of a mutant playing strategy *y *can be written as follows for *n *= 3, *T *= 2 [see [71]]:

(1) $$W(y,x)=rx^{2}+y(2rx(1-x)-c)$$

Where *c *is the cost of cooperation and *r *is the benefit received by all of the individuals if the number of cooperators is equal or above *T*. If *x *is a Nash equilibrium then the *W *should be independent of *y *(since any strategy is either cooperator: *y *= 1 or defector: *y *= 0, or any in between should get the same payoff). This condition holds only if the second part of Eq.1. equals zero [71], that is:

(2) g(x)=2rx(1−x)−c=0

Accordingly there are two solutions for *x *[71]:

(3) $$x_{1}=\frac{1}{2}+\frac{1}{2}\sqrt{1-2\frac{c}{r}},\;x_{2}=\frac{1}{2}-\frac{1}{2}\sqrt{1-2\frac{c}{r}}$$

Evolutionary stability further requires that *g*'(*x*) < 0 thus *x*_1 _is an ESS solution while *x*_2 _is an instable equilibrium. This gives the simple bifurcation diagram shown in Figure 1. in Bach *et al*. 2006 [71]. The fitness of a given strategy for any group size and for any threshold number can be written up and the equation equivalent to Eq.2. can be derived accordingly. The following general formulas can be obtained:

(4) $$\begin{array}{l} \begin{array}{lll} x^{n-1}(r-c)-c(1-x^{n-1})=0 & \text{if} & T=n \end{array}\\ (r-c)\sum_{i=n-1-j}^{n-1}\left(\begin{array}{c} n-1\\ i \end{array}\right)x^{i}{\left(1-x\right)}^{n-1-i}-c\sum_{i=0}^{n-2-j}\left(\begin{array}{c} n-1\\ i \end{array}\right)x^{i}{\left(1-x\right)}^{n-1-i}-r\sum_{i=n-j}^{n-1}\left(\begin{array}{c} n-1\\ i \end{array}\right)x^{i}{\left(1-x\right)}^{n-1-i}=0\\ \begin{array}{lll} & \text{if} & T\neq n,T\neq 1,\text{ where 1}<j<n-\text{1}\\ r{\left(1-x\right)}^{n-1}-c=0 & \text{if} & T=1 \end{array} \end{array}$$

While analytical solution might not be possible to calculate at large group sizes, numerical solutions can be obtained. Of course, the numerical solutions of this kind will not tell us which points are the stable and which points are the instable [but see ref. [72]]. To check the stability of our fixed points we used an individual based simulation of the model (details see below). The numerical results and the results of the simulations are depicted on Figure 3.

We also substitute the strict deterministic step-wise benefit function with a sigmoid probabilistic function.

(5) $$P=\frac{1}{1+e^{\left(-s)*(cn-T\right)}}$$

Where *P *is the probability that the public goal is reached, which depends on the steepness of the function (*s*), the number of cooperators in the group (*cn*) and the threshold value (*T*).

### Individual based simulation of the model

We model the evolutionary dynamics in a finite size population consisting of *N_all _*individuals (10,000 and 12,500 individuals). We set up two different scenarios in which the population structure is modelled by the two extremes. On the one part individuals have no fixed partners in a well-mixed model. In every time step both the composition of cooperating groups and competition neighbourhoods change (*n*, we increase the number of cooperators up to 50 on Figure 3.b), where partners randomly chosen from the entire population, so the interaction environment of the individual is well-mixed. In the case of spatially explicit model individuals occupy the grid points of a regular lattice (*N *× *N *= *N_all_*), with toroidal boundary conditions, and the focal individual has a constant interaction environment, its immediate neighbourhood. The neighbourhood in both cases defines both the cooperating and the competing group for the focal individual (*n*), which is 4 (von Neumann neighbourhood) group members in the spatially explicit model. The simulations were run for a given number of update steps with asynchronous updating. An update step consists of a game played by the group, individual competition, group competition if occurred (see Figure 1), and mutation.

During competition step, we use a pair-wise comparison update rule [6], which is the finite population analogue of the replicator dynamics (called the *imitate the better*, for further details see Additional file 1), in which an individual adopts the strategy of a randomly chosen neighbour with a probability proportional to the fitness difference, only if this difference is greater than zero.

$$p=\frac{W_{rival}-W_{focal}}{W_{\max}-W_{\min}}$$

We analyze multiple competition update scenarios (see Additional file 1 for details).

We also model territorial group behavior, in which case groups compete with each other for territories (sites). Groups composed of *n *individuals living in the same site. The rivals could be neighbouring groups in the spatial population (Moore neighbourhood, that is 8 closest sites) or groups randomly chosen from the population in the well-mixed case. Groups are compared according to the average group payoffs, and the successful group entirely replaces all members of the loser one. We calculate the average payoff of the group ($\bar{W}$) as the arithmetic average of the payoffs of all group members, that is the participation in the group competition is compulsory.

$$\bar{W}=\frac{\sum_{j=1}^{n}W_{j}}{n}$$

The chance for each group to occupy the focal site is given as described above in the competition rules, but using average group payoffs.

Next we introduce voluntary participation in both of the two group actions, and individuals have two continuous, evolving traits. Besides, *x *that defines the propensity to cooperate in the PGG, we introduce *a*, which describes the individual's propensity to participate in the territorial group defense action. The marginal values are also 0-1. If *a *is high, the individual will participate in the group competition with high probability, whereas if it is low or 0, it will never. As the group defense is considered as an act of cooperation here, it involves a cost *C(a) *for those who participated. The average group payoff is now calculated as the average of the payoffs of those group members, who participate in the group competition. Groups then compared by their average group payoffs according to the above described competition rules.

To ensure evolution, after the competition steps, mutation can occur in the traits of the focal individuals with the chance 0.01. The mutant's trait is drawn from a normal distribution, with the original trait value as a mean and with a given variance (0.01). The trait can only be between 0 and 1. After individual competition, only the focal individual's traits can mutate, after group competition in all members of the focal group has a chance for mutation to occur with the same probability.

The points on figures were calculated as the average of the last 1 M (10^6^) update steps of multiple iterated simulation results (for Figure 4 15 and for Figure 5 3 repetitions, with insignificant variation between the repetitions). The length of the simulation was determined by preliminary simulations, that is 1,000 M, 625 M and 312.5 M update steps for Figure 3, 4 and 5 accordingly. Graphs on Figure 5.a-d were obtained by plotting the trait distribution of the population in every 100th update step for 25,000 steps.

## Authors' contributions

GB and SzSz contributed equally to the research and the preparation of the article. GB and SzSz participated in the design of the study and in the analysis and interpretation of the results. SzSz analyzed the analytical model. GB constructed and carried out the analysis of the IBM model. All authors read and approved the final manuscript.

## Supplementary Material

Additional file 1 **Supplementary Information**. The file contains additional simulation results and their interpretation. Additional simulations were made with different update rules, different population structure models. Here we also show and analyze some representative time series simulation results. Text and graph files are included. Click here for file
