# Supplementary material for: Beneficial laggards: multilevel selection, cooperative polymorphism and division of labour in threshold public good games
Source: BMC Evol Biol. 2010 Nov 2;10:336. doi: 10.1186/1471-2148-10-336 (PMC2989973; doi:10.1186/1471-2148-10-336)
Supplement: Additional file 1 — Supplementary Information. The file contains additional simulation results and their interpretation. Additional simulations were made with different update rules, different population structure models. Here we also show and analyze some representative time series simulation results. Text and graph files are included. [file 1471-2148-10-336-S1.DOC]

**Beneficial laggards: multilevel selection, cooperative polymorphism and division of labour in Threshold Public Goods Games**

Boza, G.^1,3^ & Számadó Sz.^1,2^

^1^ Eötvös Loránd University

^2^ HAS Research Group for Theoretical Biology and Ecology

^3^ Collegium Budapest, Institute for Advanced Study,

Hungary, Budapest

**Supplementary Methods & Equations**

***Individual based simulation of the model***

We model the evolutionary dynamics in a finite size population consisting of *N_all_* individuals (10,000 individuals for simulations with individual competition and 12,500 individuals for the combined model). The simulations were run for a given number of update steps with asynchronous updating as demonstrated on Fig.2. An update step consisted of a game played by the group, individual competition, group competition if occurred (see Fig. 2), and mutation. The points on figures were calculated as the average of the last 1 M update steps of multiple iterated simulation results (for Fig. 4 fifteen and for Fig. 5 three repetitions, with insignificant variation between the repetitions). The length of the simulation was determined by preliminary simulations, that is 1,000M, 625M and 312.5M update steps for Fig. 3, 4 and 5. Graphs on Fig. 5.e-h were obtained by plotting the trait distribution of the population in every 500th update step for 25,000 steps.

*Population structure.*

We set up two different scenarios in which the population structure is modelled by the two extremes. On the one part individuals have no fixed partners in a well-mixed population model. In every time step both the composition of cooperating groups and the competition neighbourhood change: partners randomly chosen from the entire population, so the interaction environment of the individual is well mixed. In the case of spatial model individuals occupy the grid points of a regular lattice (*N* × *N* = *N_all_*), with toroidal boundary conditions, and the focal individual has a constant interaction environment, its immediate neighbourhood. The neighbourhood in both cases defines both the cooperating and the competing group for the focal individual, which can be 4 or 8 group members (up to 50 in the well mixed model on Fig. 3). We use asynchronous updating method, every generation consists of *N_all_* updates, and in every update step we choose one individual from one site, and update its payoffs and reproduction. We model the spatial set up in two ways. In the basic scenario, individual competition occurs between members of the same group (type A). In the second scenario, the focal individual competes with a given number of randomly chosen individuals from the neighbourhood. This means, that competition can occur between group members, or between individuals from the neighbouring sites (type B). We used both the von Neumann (4 closest neighbours) and the Moore neighbourhood (8 closest neighbours) for our simulations.

*Individual selection.*

A randomly chosen individual’s fitness is updated according to the composition of its cooperating group. In the case of individual selection the fitness of an individual is compared with the member(s) of the competing group. We used multiple competition update scenarios.

(1) In the basic set up, we used the *imitate the better* (ITB) update rule [1,2], which is the finite population analogue of the replicator dynamics [1] and as a result it is the most frequently used update in individual based simulations [1,3]. Here a randomly chosen rival individual from the competing group and the focal individual compete for the focal site. The probability of the rival individual taking over the focal site or in other words the rival’s strategy being imitated by the focal individual (*p*) is proportional to the difference of the individual payoff (*fd*) compared to the maximal possible payoff difference of the game (*fm*), provided that the difference is positive and with zero probability otherwise.

$$p=\frac{\Delta f}{f_{md}}$$

where

$$\Delta f=f_{rival}-f_{focal}$$

$$f_{md}=f_{\max}-f_{\min}$$

which is

$$f_{md}=c+b$$

(2) In the *proportional* (PR) update rule [1,4], all the members of the competing group and the focal individual compete for the focal site. The chance for an applicant to take over the focal site is proportional to its fitness:

$$p_{i}=\frac{f_{i}}{\sum_{j=1}^{l} f_{j}}$$

where the denominator is the summed payoff of all applicants (*l*= focal + neighbours).

(3) In the *imitation* (IM) rule [2,4,5], a randomly chosen rival individual from the competing group and the focal individual compete for the focal site. The probability of the rival individual taking over the focal site, or in other words the rival’s strategy being imitated by the focal individual (*p*) is proportional to the individual’s payoff (*fd*) compared to the summed payoff of the rival and the focal individuals.

(4) In the *pair wise comparison* (PC) update rule [2,6], a randomly chosen individual and the focal individual compete for the focal site according to payoffs. The rival individual takes over the site with the probability $p=\frac{1}{1+\exp(-w\Delta f)}$, where $\Delta f=f_{rival}-f_{focal}$, and *w* is the strength of selection, which in the current model was set to 1.

(5) During the *death - birth* (DB) update scenario [2,5], a random individual is chosen to die. All the neighbouring individuals in the competing group have the chance to place an offspring to the focal site proportional of their payoffs.

(6) In the *birth -* *death* (BD) update [2,5], an individual is chosen from the entire population to reproduce proportional to the payoff. A random neighbour of this individual dies, and the offspring occupies this site. Because of computational limitations, we used 10% random sampling of the population.

(7) In the *best takes over* (BTO) update rule [1,4], the focal individual and its competing group compete for the focal site. The one with the highest payoff places an offspring to the site.

*Introducing group selection with compulsory participation.*

We also model territorial group behaviour, in which case groups compete with each other for territories (sites). Groups composed of *n* individuals living in the same site. The rivals could be neighbouring groups in the spatial population (Moore neighbourhood) or groups randomly chosen from the population in the well-mixed case. Groups compared according to the average group payoffs, and the successful group entirely replaces all members of the loser one. We calculate the average payoff of the group as the arithmetic average of the payoffs of all group members, that is the participation in the group competition is compulsory.

$$\bar{F}=\frac{\sum_{j=1}^{n} f_{j}}{n}$$

The chance for each group to occupy the focal site is given as described above in the different competition rules, but using average group payoffs.

*Introducing group selection with voluntary participation.*

In the case of voluntary participation in both two group actions, individuals have two continuous, evolving traits. Besides, *x* that defines the propensity to cooperate in the PGG, we introduce *a*, which describes the individual’s propensity to participate in the territorial group defence action. The marginal values are also 0-1. If *a* is high, the individual will participate in the group competition with high probability, whereas if it is low or 0, it will not. As the group defence is considered as an act of cooperation here, it involves a cost *C(a)* for those who participated. The average group payoff is now calculated as the average of the payoffs of those group members, who participate in the group competition. Groups then compared by their average group payoffs according to the above described competition rules.

*Mutation.*

To ensure evolution, after the competition steps, mutation can occur in the traits of the focal individuals with the chance 0.01. The mutant’s trait is drawn from a normal distribution, with the original trait value as a mean and with a given variance (0.01). The trait can only be between 0 and 1. After individual competition, only the focal individual’s traits can mutate, after group competition in all members of the focal group has a chance for mutation to occur with the same probability.

For our simulations and plots we used the following softwares: Borland Delphi 7, Mathematica 5, R, Gnuplot.

**Supplementary text**

Figure S1 depicts the results when group defence is voluntary as a function of the two kinds of costs and the initial number of the cooperators in the PGG for the seven different kinds of updates. The updates are arranged from the left to the right as an increasing function of their propensities to favour cooperation (BD, ITB, PC, IM, BTO, PR, DB). The upper two rows show the well mixed case (*a_i_*=0 and *a_i_*=1 respectively), while the lower two ones show the results of the spatial model (*a_i_*=0 and *a_i_*=1). At the left part of the graph, in the cases it is harder to maintain cooperation, we observe stable levels of cooperation at lower cost values and after a sharp transition, defection. At the transition boundary, division of labour may occur. As we move towards the updates that favour cooperation at the right part of the graph, the parameter region of cooperation extends and the phenomena of division of labour vanishes. The very difference that these updates bring in (such as proportional or death-birth rules) is that the minimal allowed payoff of the individuals is 0. Accordingly, participation in the group competition action is 1, because every joining member adds a zero or non-negative value to the group payoff, so full participating groups out compete the groups with lesser participation. In other words, the total payoff of the group is increasing as a function of group participation in these cases. In contrast, in case of the previous update scenarios (such as imitate the better rule), individual payoffs can undergo 0, thus the group total payoff function can decrease if individuals with less than zero fitness join the group competition stage. Accordingly full participation is not always selected for in these cases.

The biological intuition for these results are as follows: if every individual contributes to the success of the group at the group competition stage regardless how tired or exhausted it is (i.e. increasing group pay-off as function of participation) then obviously it is worth participating and thus groups with full participation out compete less cooperative groups (see updates: PR and DB). On the other hand, if exhausted, weak or injured individuals do not increase the success of the group at the group competition stage or perhaps even decrease it (because they hazard the fighting success of the group) then full participation will be selected against when the combined costs of hunting and group defence is high. Also division of labour can be selected for in such cases. Of course if the combined costs are too high then cooperation is not stable and thus division of labour cannot evolve either. Thus we can see that division of labour is expected to evolve on the boundary of cooperative vs. non-cooperative regimes when group fitness at the group competition stage is not necessarily a monotone increasing function of participation.

Note that even those updates that favour full participation at the group competition stage (PR) born out our main conclusion that the group hunting trait is polymorphic at the non-zero equilibria (i.e. dots range in colour from lighter shade of red to orange, except when cooperation is cost-free, *C*(*x*)=0, when we observe full cooperation, i.e. dark red coloured bubbles).

Our studies show that the precise choice of mutation rate and variance, in which through we can manipulate the stochasticity driven distribution of the population, can bring different results. When the average distribution of mutants in the population is low, and the mutation variance is kept at medium levels, divergent strategies and short term dimorphism in *x* emerged in the population, however still no long term stability was observed.

The lower row in Fig. S1 shows the results of the spatial model. The positive effect of spatial structure on the evolution and stability of cooperation is more pronounced with the update rules that disfavours cooperation (ITB and PC). The strongest beneficial effect of the spatial structure on the level of cooperation is observed when individuals compete within the group (type A). In these cases no division of labour occurred in our simulations.

We get different results if the focal individual competed all the individuals surrounding its site, including own group members and members of the group from neighbouring sites (type B) (Fig. S2 row c). Space has still a positive effect on the average outcome of our simulations compared to the well mixed case, but not as intense as in the previous model (i.e. type A, Fig. S2 row b). Cooperation is stable only at intermediate or lower cost values, and some of our simulations showed the division of labour in this model (see also Fig. S3.G).

We also studied the effect of different threshold levels (Figure S2). While high threshold levels select for high level of cooperation (as expected from the results of the IBM with only individual level selection), it has a very narrow basin of attraction. On the other hand cooperative equilibria have a larger basin of attraction with low threshold levels, but we also observe lower cooperation levels for higher cost values. Division of labour appears for any intermediate *T* values in the well mixed model, as indicated by the half size orange bubbles. Our analysis shows that spatial population structure extends the parameter space that leads to cooperative regime for every threshold value.

Figures S3 and S4 presents the dynamics of our simulations that lead to the different equilibrium points of the system when *T*=3, with imitate the better update rule (Fig. S3) and proportional update rule (Fig. S4). We show results from the well mixed model (S3.A-D, S4.A-B), and from the model with spatial population structure (S3.E-G, S4.C). One can follow the average cooperational trait values of the population as time series plots (panels **a**), as well as the group composition distribution (panels b), where group composition refers to the number of cooperators in a group at a given update step. Starting from medium initial trait values, depending on the parameters of the simulation, either both traits (*x* and *a*) increase in time towards high stable levels of cooperation (S3.A) or evolve to defection (S3.C). In between, for a narrow range of parameters division of labour appears in the cooperating tasks (S3.B). One can see the continuous transition of group compositions towards the optimal cooperator: defector ratio. For easier understanding we calculated the average success of the given group compositions (panel c). One can follow that the optimal group composition becomes the most successful one shortly after the beginning of the simulation. Also because of mutation, the least successful groups are produced continuously, which shows up on the graphs as strong fluctuations (for example group Coop. Nr. 2 on S3.C). The transition to the optimal group composition can be quick (S3.D) or a more slowly and more stochastic process (S3.B), and also in the model with spatial populations structure, the fixation takes more time (S3.E-G). When we introduce spatial population structure into the model, one can note that the transition from the initial state to the fix point takes place in several localities in the population. There are separately evolving clusters, with different phases of evolution and this makes the process slow.

One interesting phenomena can be seen on Figure S3.B. At the point in time where division of labour, that is the dimorph absorbing states stabilize, the equilibrium ratio and average success of the hunting group types 4 and 3, and 5 and 2 become the same. As the optimal number of cooperators is 3, the most successful group type is the 3, but as cooperative decision is a stochastic process, the group often ends up with 4 or 2 cooperators. Group type 4 is almost equally successful, as it can beat any other group, except for the type 3. Group type 2 ends up without acquiring the benefit, and type 5 has too many cooperators, so the group total payoff is relatively small, so these two can be out competed by the superior types. When the system reaches the mutation-selection balance the formation and disappearance of weaker group types (i.e. groups consisting of 1, 2 or 5 cooperators) is in equilibrium.

Also interesting to see, that on Figure S3.C evolution first drives the system towards higher *x* values, only later in time it takes a turn towards full defection. Before the spread of full defecting mutants, which in this case destabilize the system, group types that are above the threshold enjoy the benefit of cooperation for a short term.

Similar pattern can be seen on Figure S3.G, when division of labour evolves in the type B spatial model. The system first evolves towards full cooperation in both group actions, but in this environment, cost-reducing groups can invade, and this leads to the optimal group composition state.

In both above mentioned cases, the success of the given group type depends on the surrounding cooperative environment, and the migration of the different individuals (more or less cooperative individuals) between the different groups. This holds for the well mixed model and the model with type B spatial population structure. The lack of migration between groups explains why we don’t see this kind of dynamic in the model with type A spatial population structure.

In the model with proportional update rule (Figure S4), one can see that the average success of the group types is always low. This comes from the properties of the update rule. The optimal group type has the highest chance to win the competition, but the least favourable type also has some significant chance. This makes the selection weak, and group types appear in the population a proportional manner according to the relative payoff differences.

**References**

1. Hauert C, Doebeli M: **Spatial structure often inhibits the evolution of cooperation in the snowdrift game.** *Nature* 2004, **428**, 643–646.
2. Ohtsuki H, Nowak MA: **The replicator equation on graphs**. *J. Theor. Biol.* 2006, **243**, 86–97.

1. Pacheco JM, Santos FC, Souza MO, Skyrms B: **Evolutionary Dynamics of Collective Action in N-person Stag-Hunt Dilemmas.** *Proc. Roy. Soc. London B* 2009, **276**, 315-321.
2. Hauert C: **Effects of space in 2x2 games**. *Int. J. Bifurcation and Chaos* 2002, **12**, 1531-1548.
3. Számadó Sz, Szalay F, Scheuring I: **The effect of dispersal and neighbourhood in games of cooperation**. *J. Theor. Biol.* 2008, **253**, 221-227.
4. Szabó Gy, Tőke Cs: **Evolutionary prisoner's dilemma game on a square lattice.** *Phys. Rev.* 1998, **58**, 69-73.

**Supplementary Figure(s) and Legend(s)**

S1


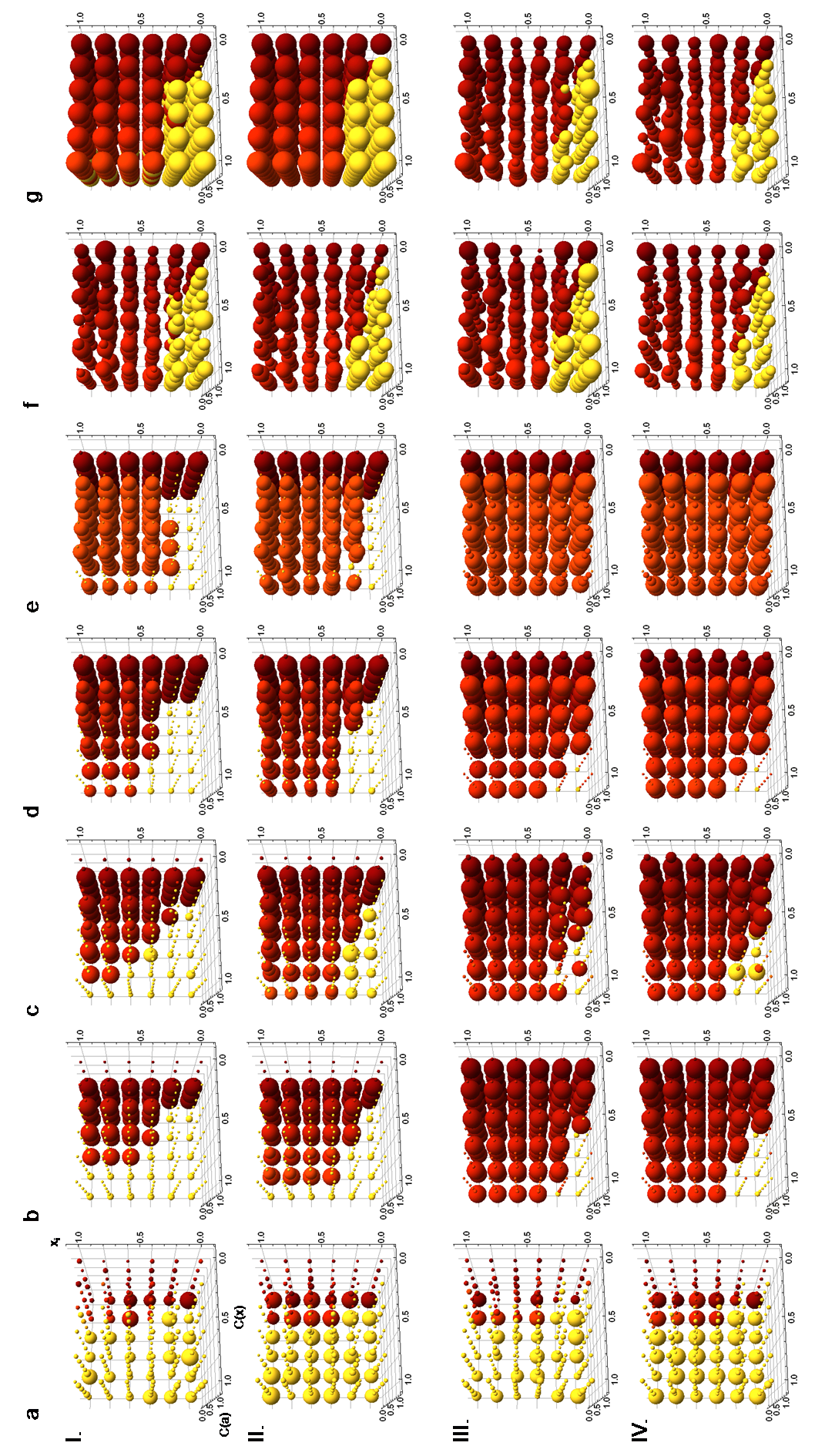


**Figure S1** The effect of different update techniques on the evolution and stability of *n*-person PD with voluntary participation. The listed different updates favour cooperation in a growing series, starting from the least favouring: (**a**) birth-death update (BD), (**b**) imitate the better (ITB), (**c**) pair wise comparison (PC), (**d**) imitation (IM), (**e**) best takes over (BTO), (**f**) proportional (PR) and (**g**) death-birth (DB) update rules. Each bubble represents the result of one simulation in a population of 12,500 individuals, and the simulations were run for 312.5 M update steps. In the first two rows (I.-II.) the population is well mixed, and we started the simulations with no initial tendency to join the group competition action, that is *a_i_* = 0 (I.), and with full initial participation *a_i_* = 1 (II.). In the last two rows, the population structure is spatial (III.-IV.), and *a_i_* equals 0 (III.) and 1 (IV.). The size and colour representations of the bubbles are the same as for Fig. 4.

S2


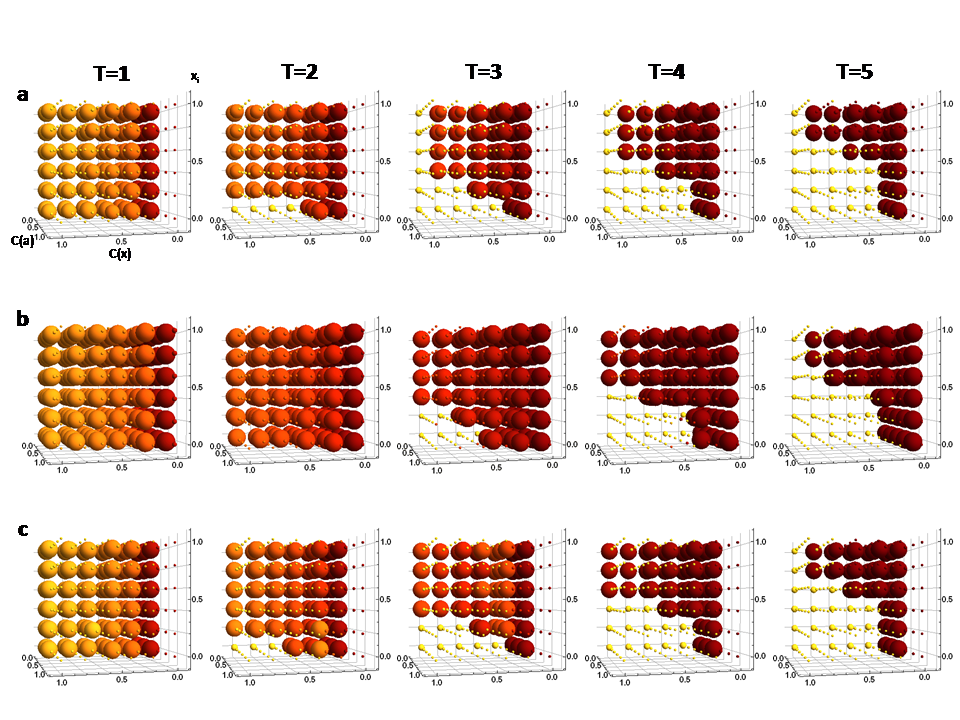


**Figure S2** The comparison of the results with different threshold levels, population structures and interaction neighbourhoods with imitate the better update rule (ITB). The first row shows the results from the well mixed model (**a**), the second and third rows are for the results with spatial population structure (**b.-c.**). The representations of the bubbles are the same as described in the main text. The model details are the same as described in the main text, with *a_i_*=1. In both of spatial models, we modelled 2,500 sites on the regular 50*50 grid occupied by a group. For simulating the spatial neighbourhood, we used two different set ups. The first one is described as type A (**b**), where the focal individual competes only with its group members (the same as in the main text). We analyzed a second set up as well, called type B (**c**), in which case individual competition occurs within the Moore neighbourhood, with a randomly chosen individual.

S3/A.


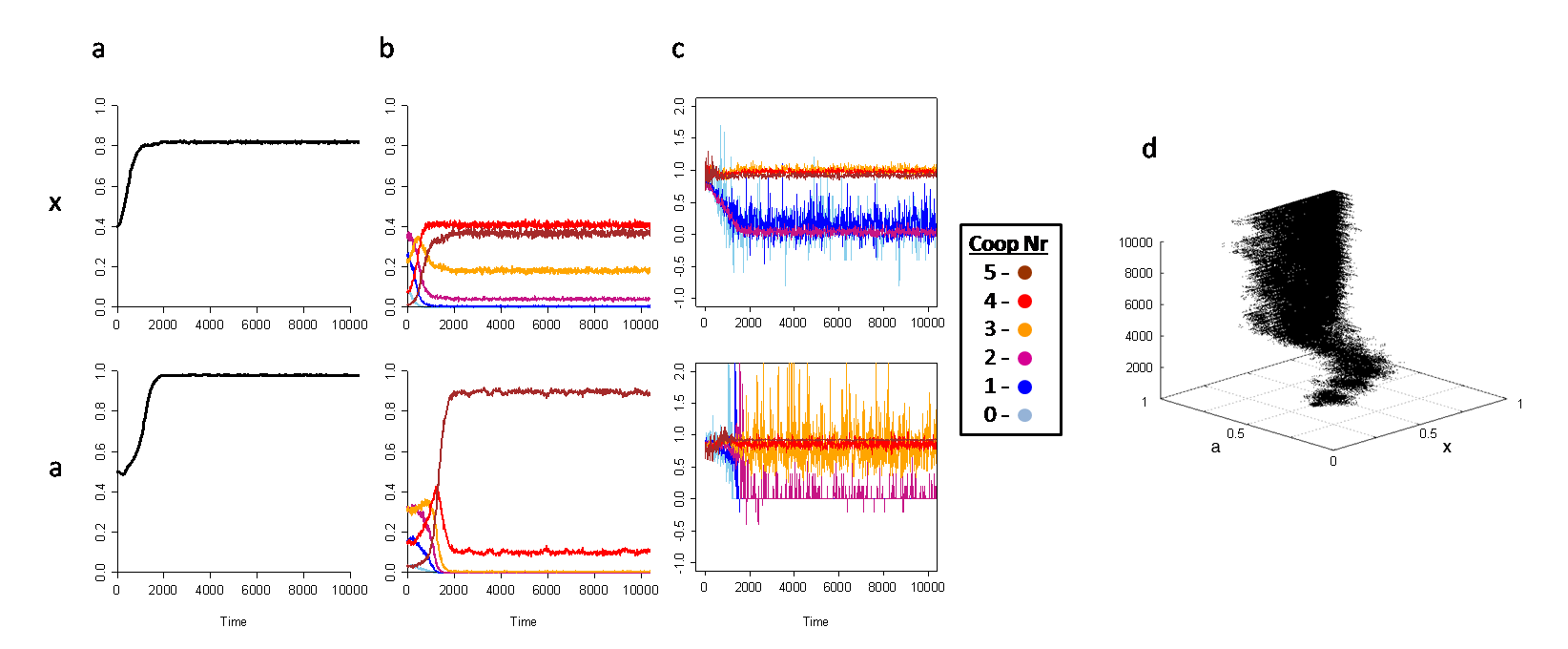


S3/B.


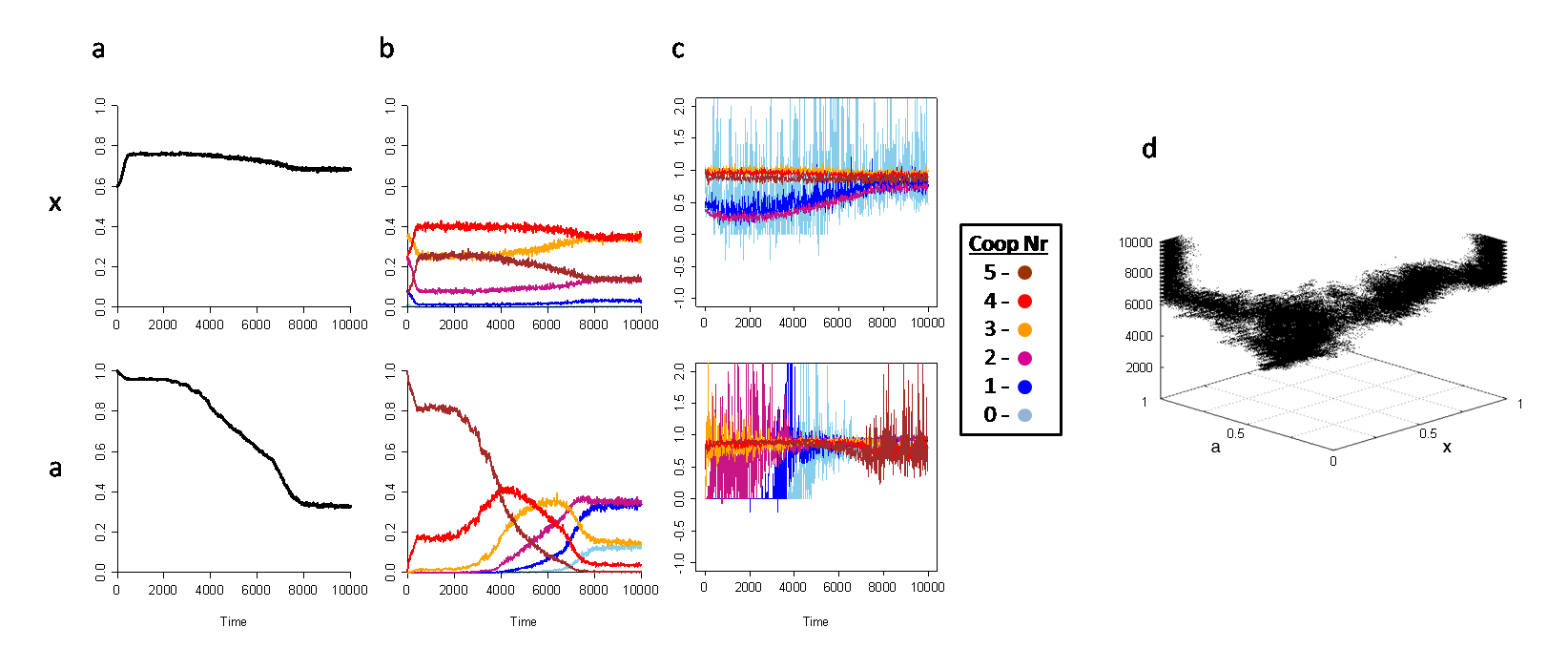


S3/C.


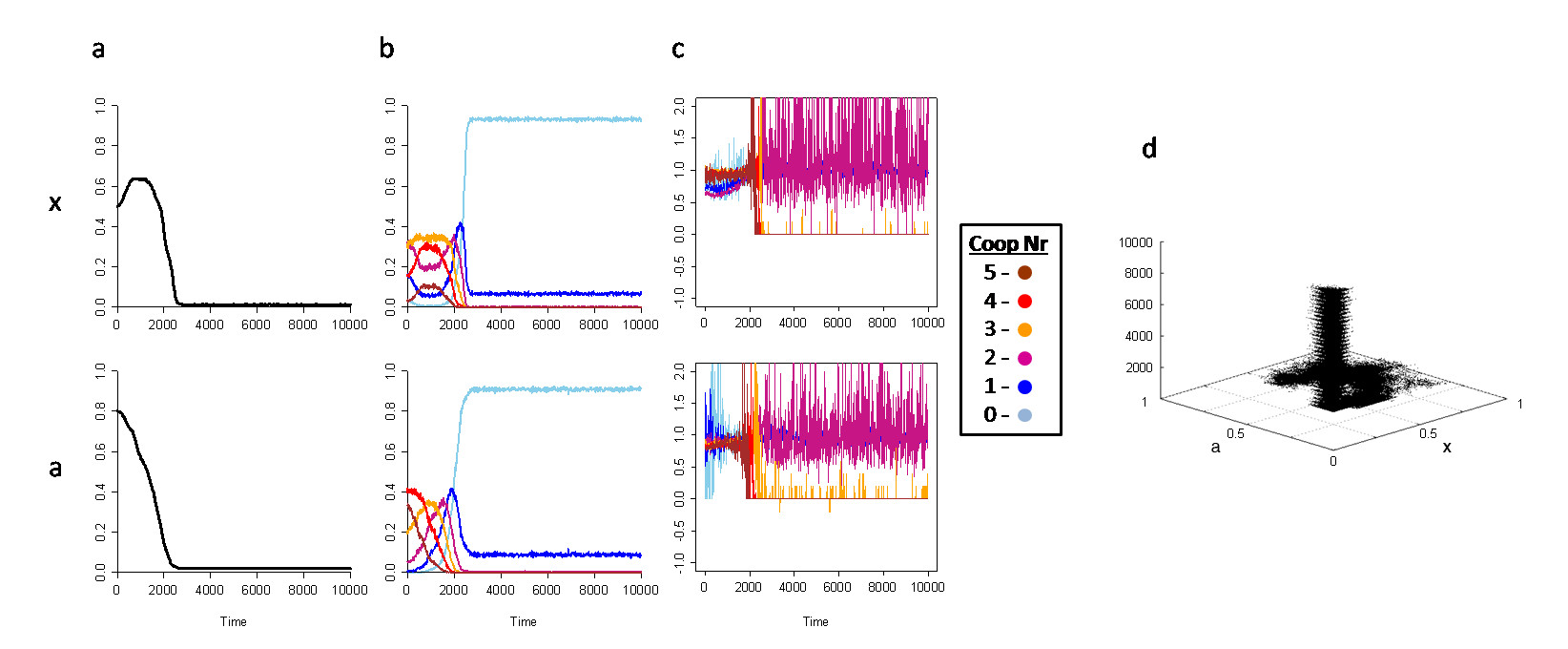


S3/D.


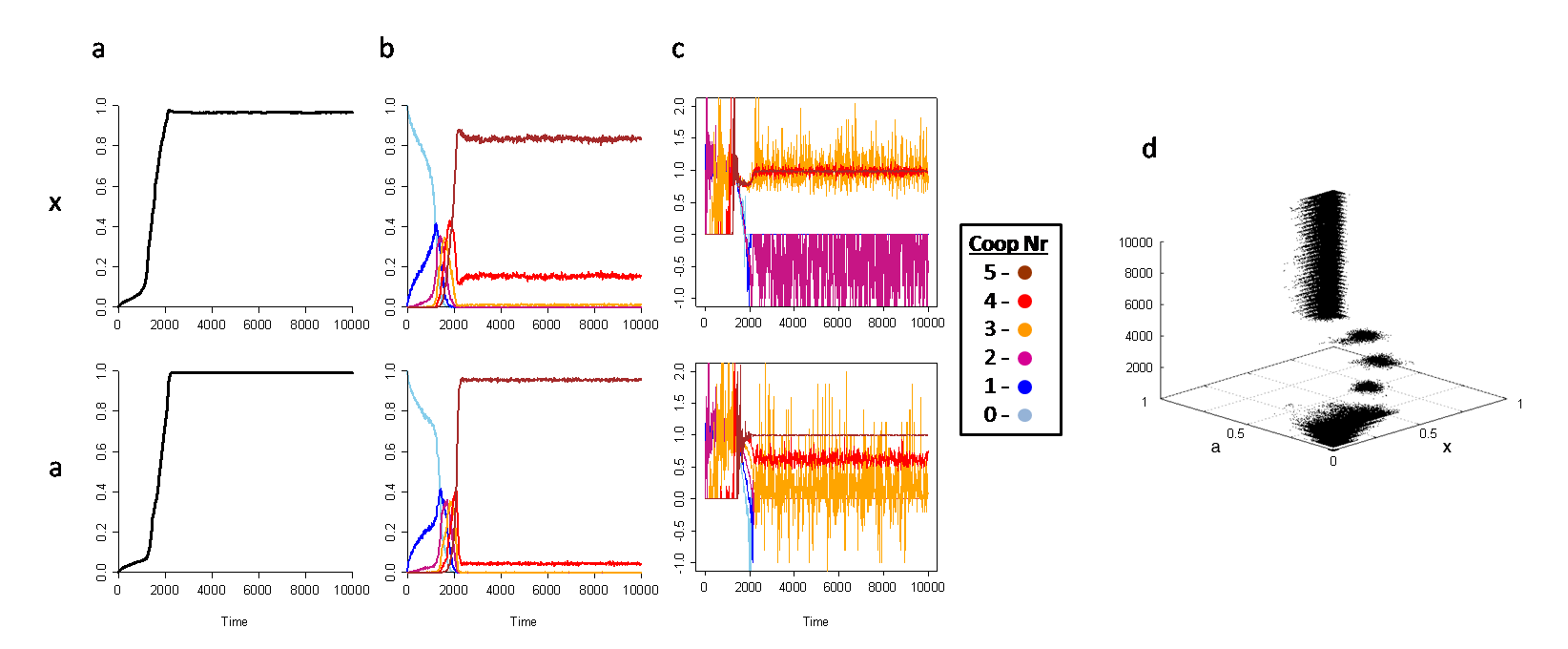


S3/E.


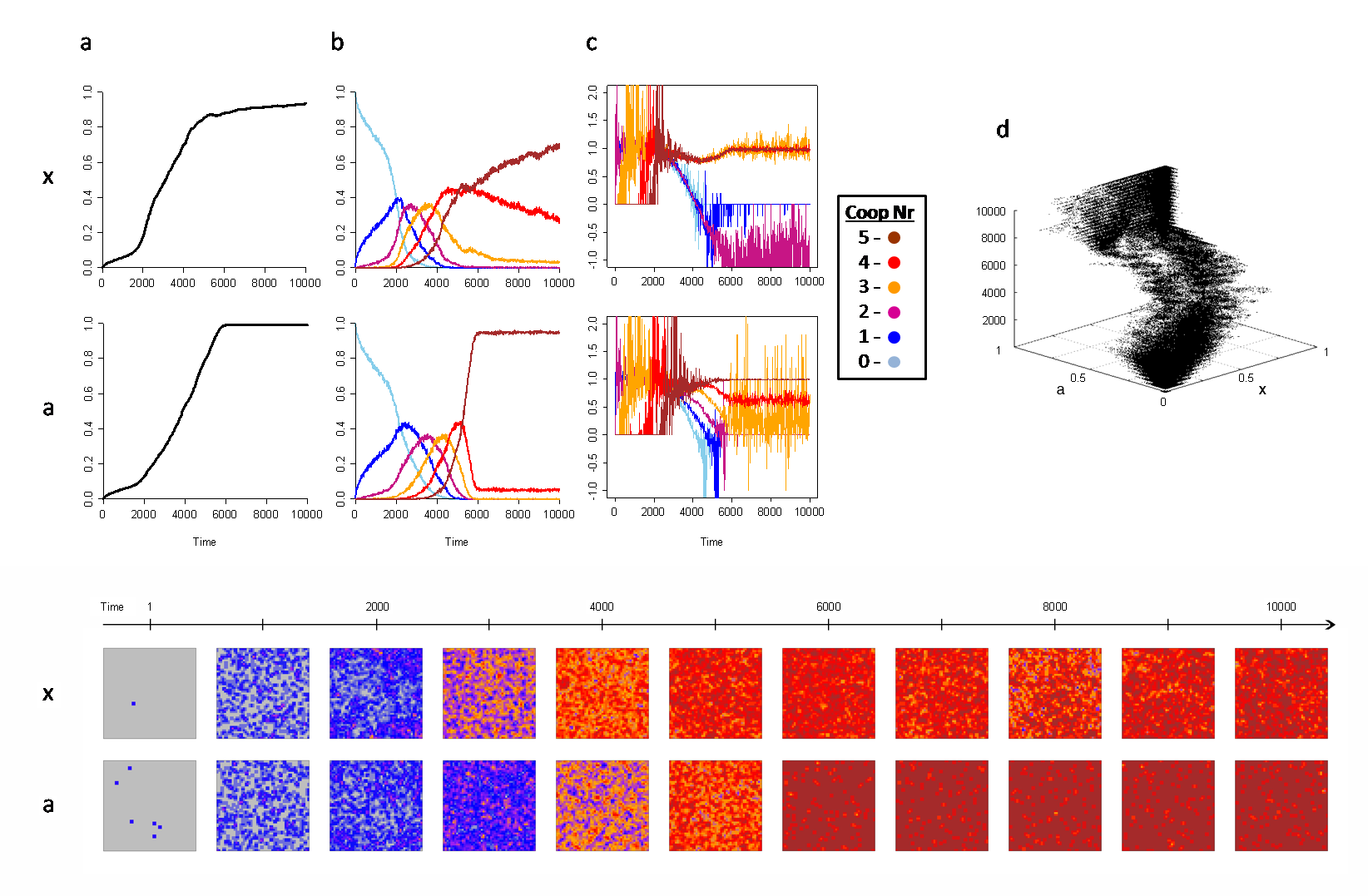


S3/F.


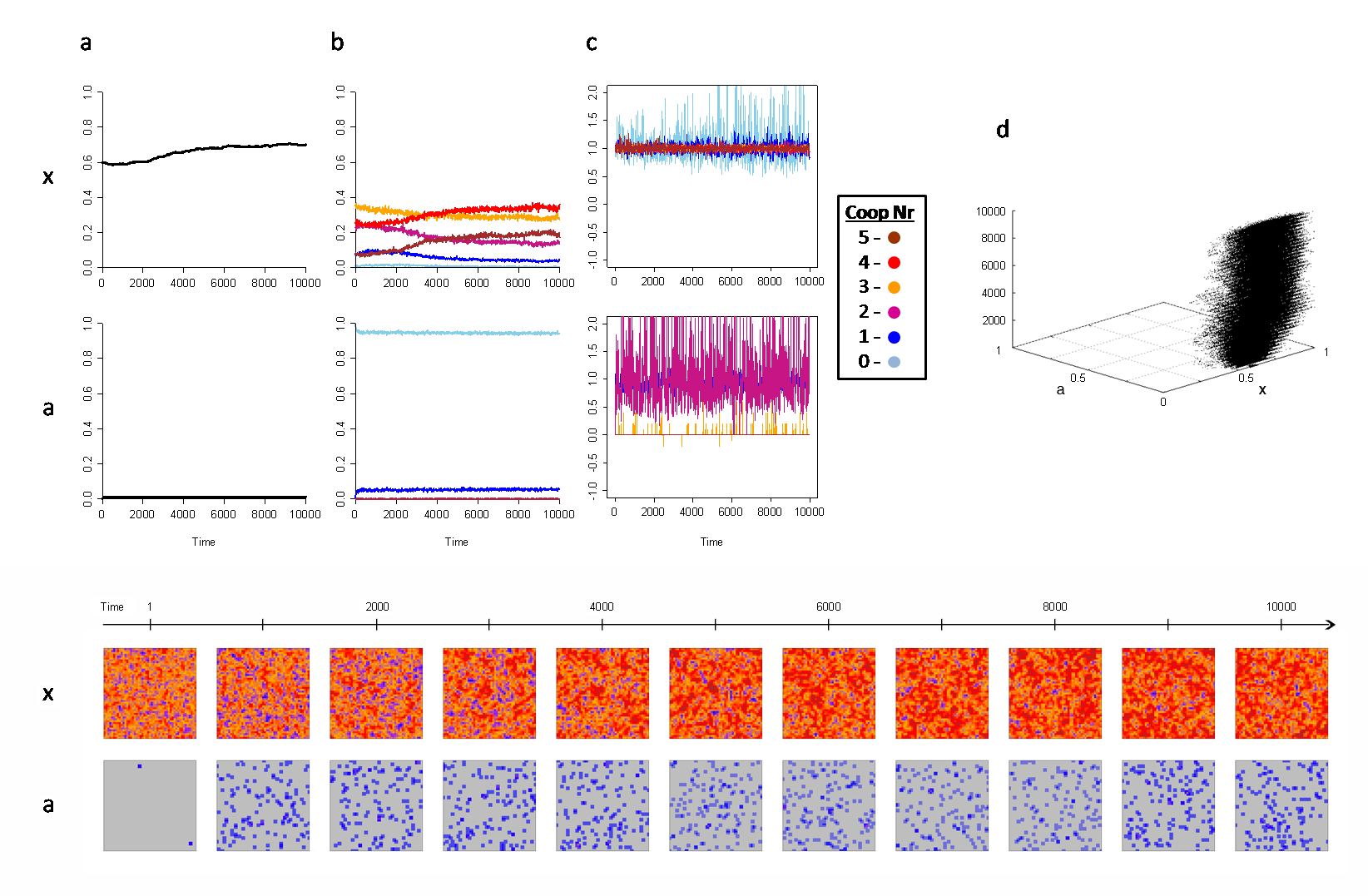


S3/G.


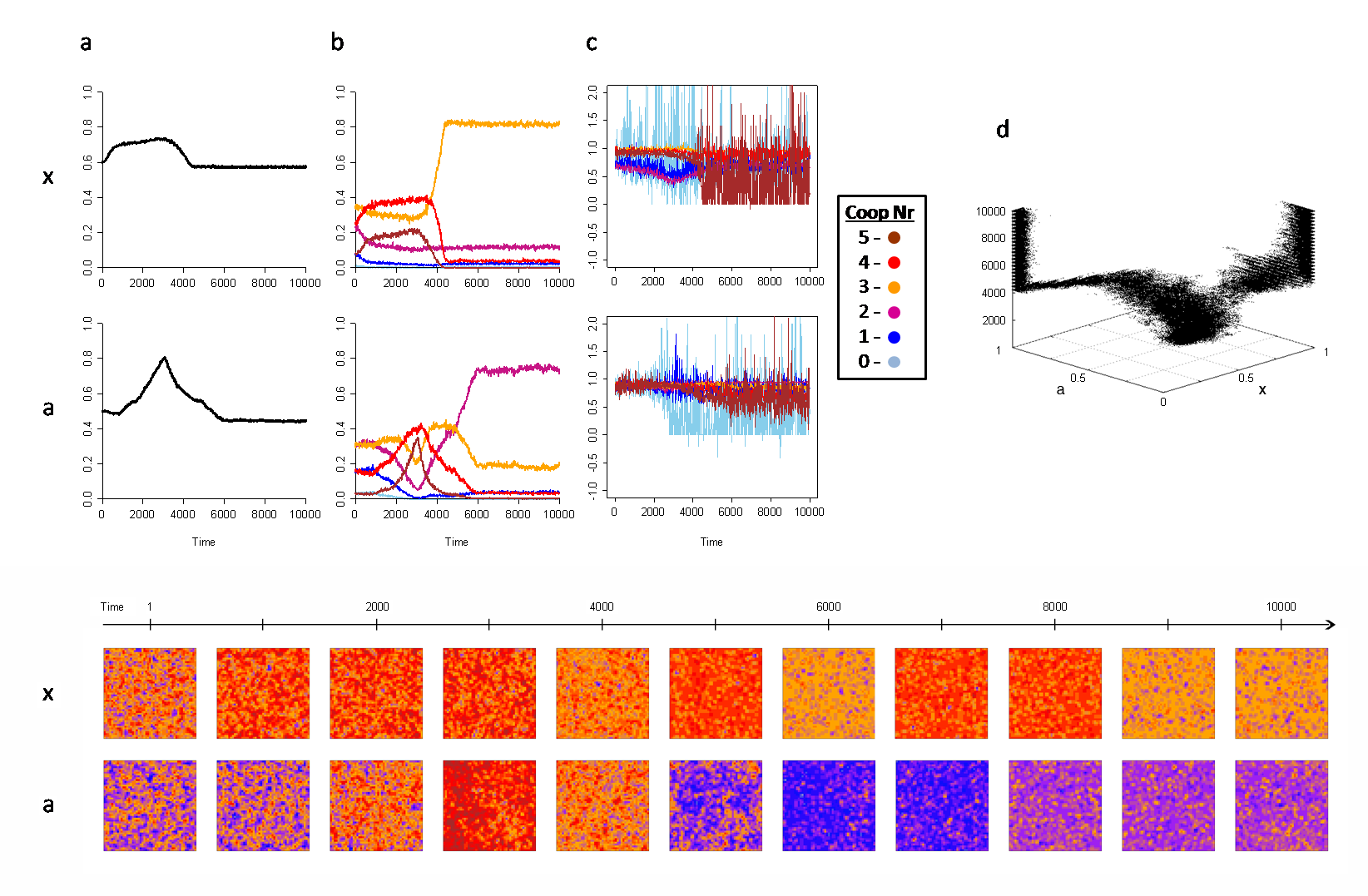


**Figure S3** Simulation results from individual based model with imitate the better update rule (ITB). The results are shown as time series graphs from 0 to 10,000 generations (that is 125 M update steps). On each panel the first row shows the attributes of trait *x* (willingness to participate in the group hunting action) and the second row shows trait *a* (willingness to participate in the group competition). The first columns on the panels show the average trait values of the population (**a**), the next row shows the frequency of groups composed of different numbers of cooperators at that update step (**b**). The group composition is coded by the colours of the lines as shown on the side panel. Colours code goes from brown to blues as the number of cooperators decrease in the group. The next column (**c**) depicts the average success of a group type. We measure the success as the difference between the number wins and loss normalized by the group type frequency. On the last panel (**d**), we show the distribution of the two traits, *x* and *a* in the trait space.

On panels A-D we used well mixed model, and on panels E-G we used spatial population structure for our simulations. On the bottom of these later panels, the spatial distribution of the different group types is shown as snapshots at certain time intervals. Colour coding used on the snapshots is the same as described before on the time series graphs. Each pixel represents one of the 2,500 sites on the regular 50*50 grid occupied by a group. For simulating the spatial neighbourhood, we used two different set ups. The first one is described as type A (panel E), where the focal individual competes only with its group members. The other set up is called type B (panels F-G), in which case individual competition occurs within the Moore neighbourhood. For our simulations we used the following parameters:

S3/A.: (C(x)= 0.4; C(a)= 0.2; *x_i_*= 0.4; *a_i_*= 0.5; well mixed population);

S3/B.: (C(x)= 0.6; C(a)= 0.2; *x_i_*= 0.6; *a_i_*= 1; well mixed population);

S3/C.: (C(x)= 0.8; C(a)= 0.2; *x_i_*= 0.5; *a_i_*= 0.8; well mixed population);

S3/D.: (C(x)= 0; C(a)= 0; *x_i_*= 0; *a_i_*= 0; well mixed population);

S3/E.: (C(x)= 0; C(a)= 0; *x_i_*= 0; *a_i_*= 0; type A spatial population structure);

S3/F.: (C(x)= 0.6; C(a)= 0.8; *x_i_*= 0.6; *a_i_*= 0; type A spatial population structure);

S3/G.: (C(x)= 0.61; C(a)= 0.21; *x_i_*= 0.6; *a_i_*= 0.5; type B spatial population structure);

S4/A.


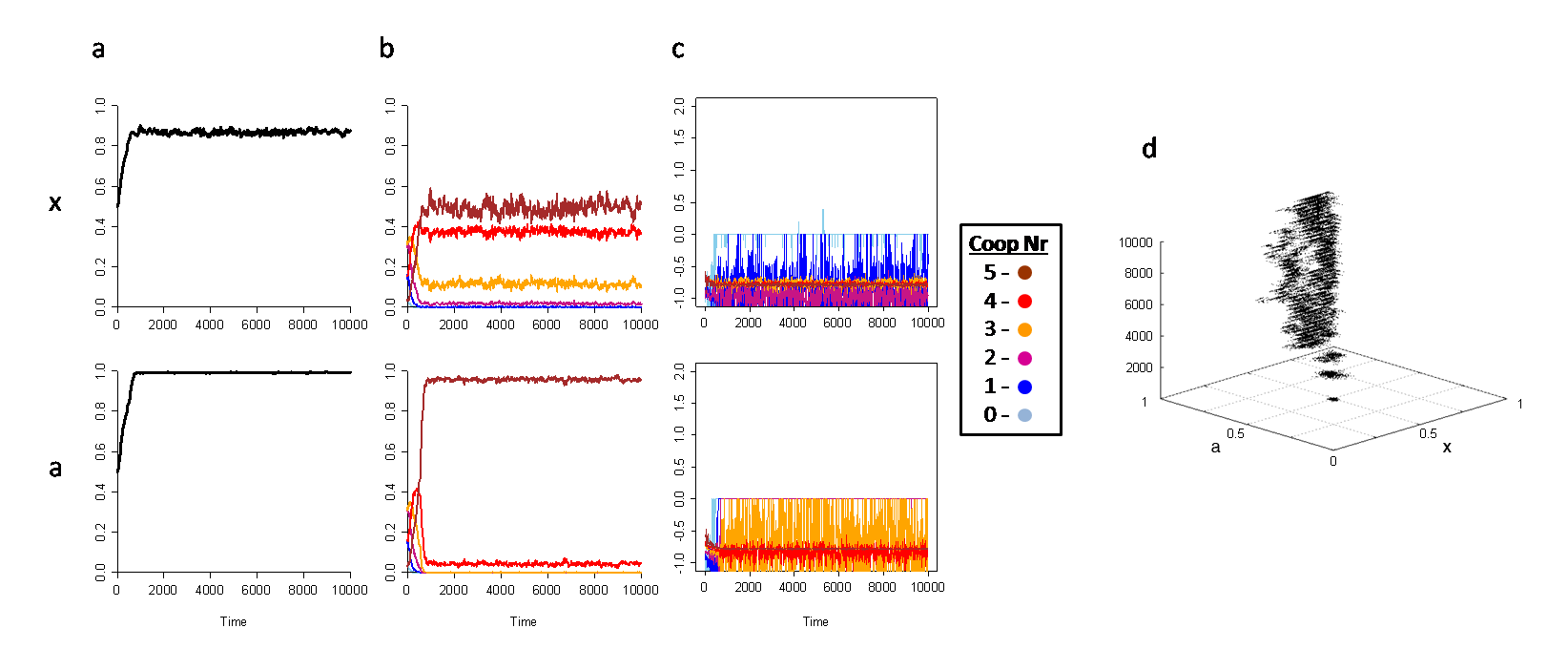


S4/B.


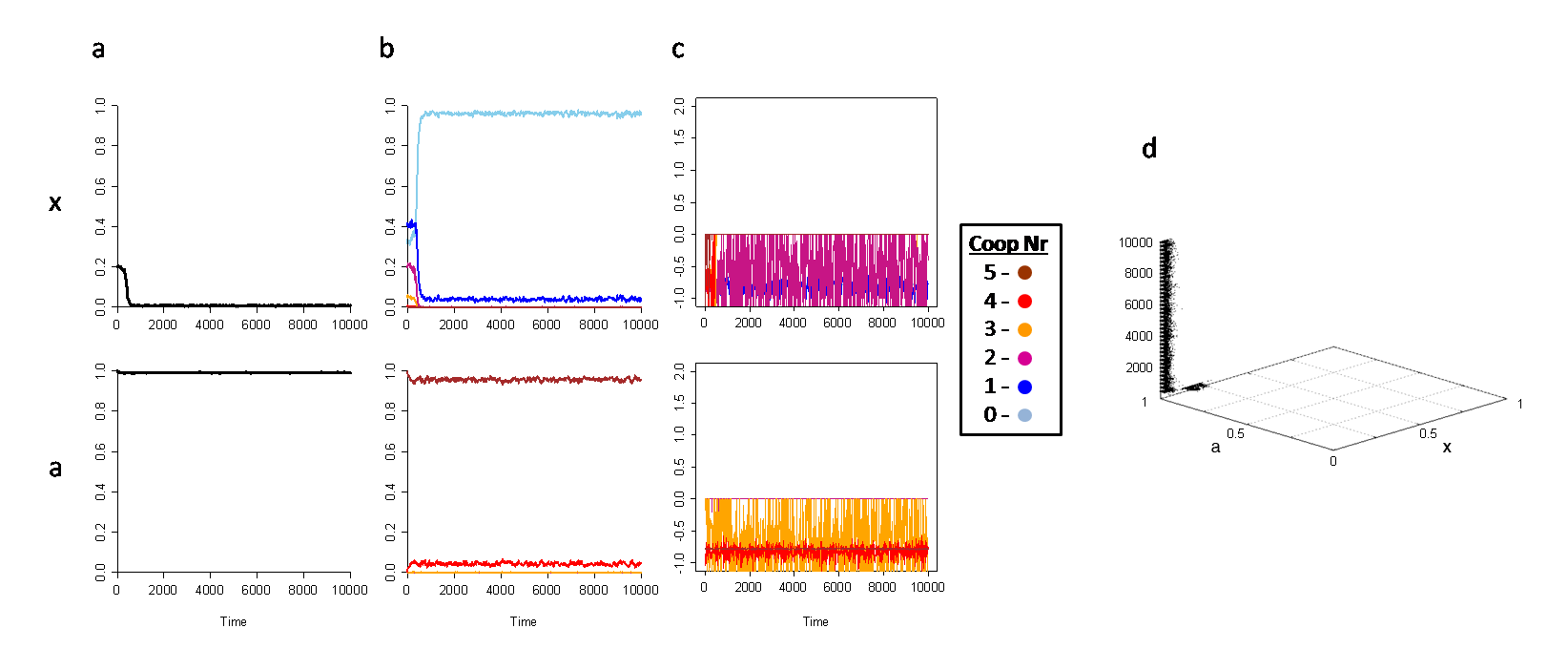


S4/C.


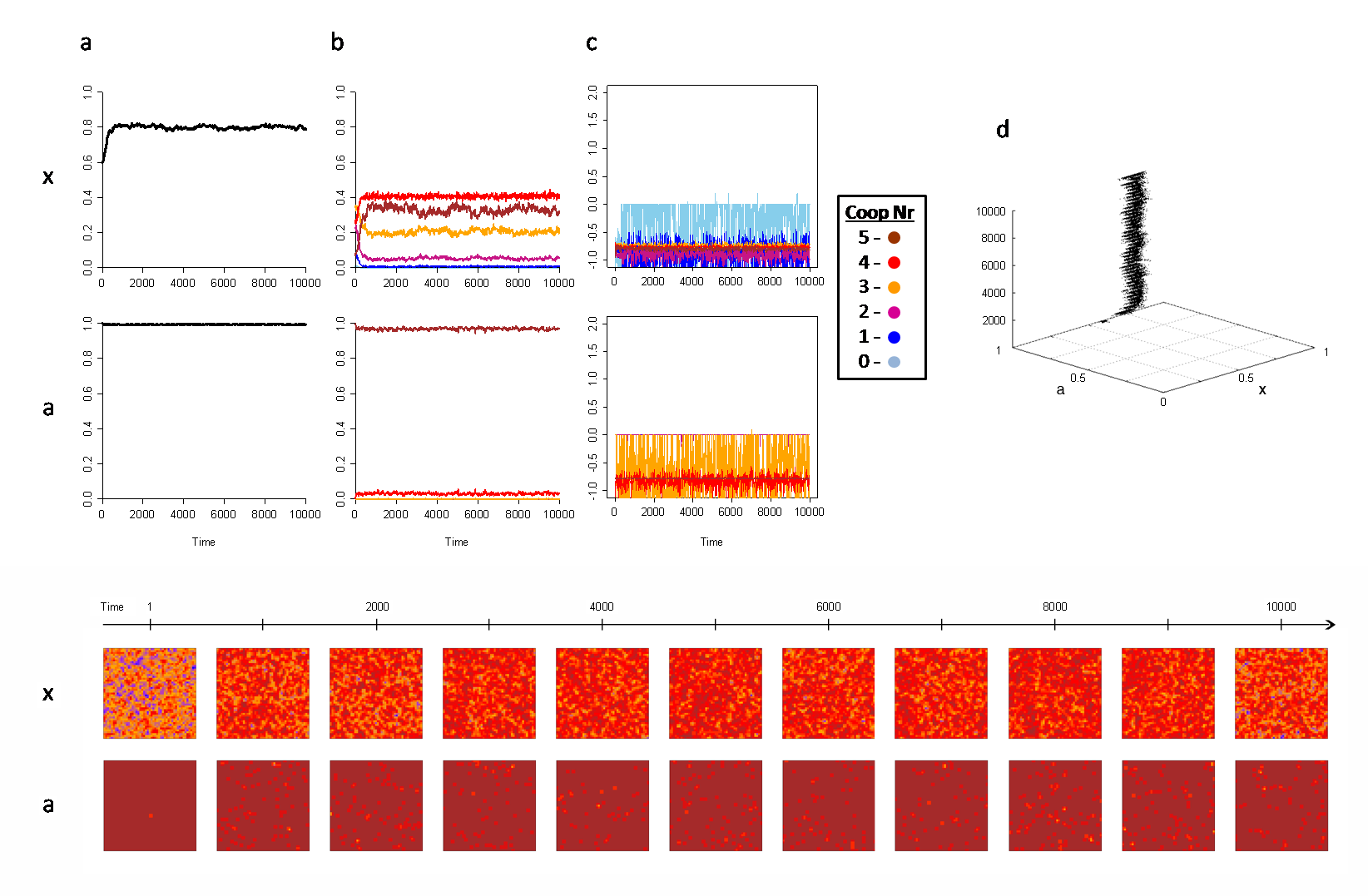


**Figure S4** Simulation results from individual based model with proportional update rule (PR). The results are shown as time series graphs from 0 to 10,000 generations (that is 125 M update steps). Panels A and B are well mixed cases, panel C. is with type A spatial population structure. Details as described before.

Parameters used for the simulations:

S4/A.: (C(x)= 0.4; C(a)= 0.4; *x_i_*= 0.5; *a_i_*= 0.5; well mixed population);

S4/B.: (C(x)= 0.8; C(a)= 0.8; *x_i_*= 0.2; *a_i_*= 1; well mixed population);

S4/C.: (C(x)= 0.8; C(a)= 0.8; *x_i_*= 0.2; *a_i_*= 1; type A spatial population structure);
